# Indoor Tanning among Sexual and Gender Minority Adolescents and Adults: Results from the 2020 Pennsylvania LGBT Health Needs Assessment

**DOI:** 10.1155/2023/3953951

**Published:** 2023-05-17

**Authors:** Christopher W. Wheldon, Joshua Zhi Hao Spradau

**Affiliations:** ^1^Department of Social and Behavioral Sciences, College of Public Health, Temple University, Philadelphia, PA, USA; ^2^College of Science and Technology, Temple University, Philadelphia, PA, USA

## Abstract

Sexual and gender minority (SGM) populations include individuals whose sexual orientation, gender identity, or reproductive development is characterized by nonbinary sexual constructs (e.g., lesbian, gay, bisexual, and transgender (LGBT) individuals). Previous research suggests that some SGM populations have higher rates of skin cancer. The purpose of this study was to assess the association of diverse SGM identities with indoor tanning, a risk factor for skin cancer, while exploring other relevant co-occurring risk factors. A secondary analysis was performed on the 2020 LGBT Health Needs Assessment collected by the Pennsylvania Department of Health. Measures included sexual orientation, gender identity, healthcare utilization, and cancer risk factors. Cisgender SGM men are more likely to use indoor tanning devices (adjusted odds ratio (aOR) = 1.79; 95% CI: 1.31–2.44) compared to other SGM subpopulations independent of sexual orientation. Indoor tanning was also associated with alcohol (aOR = 1.94; 95% CI: 1.50–2.51) and tobacco use (aOR = 1.64; 95% CI: 1.21–2.21). Findings suggest that targeted screening for skin cancer risk behaviors could accompany standard tobacco and alcohol screenings in clinical practice.

## 1. Introduction

Sexual and gender minority (SGM) populations include individuals whose sexual orientation, gender identity, or reproductive development is characterized by nonbinary sexual constructs (e.g., lesbian, gay, bisexual, and transgender (LGBT) individuals) [1]. SGM populations have been shown to have an increased risk of developing certain types of cancer compared to heterosexual and cisgender individuals [2]. Among these, skin cancer is of particular concern, as studies suggest that lifetime risk of skin cancer was 1.3–2.1 times higher among SGM males when compared to heterosexual males [3–5].

Tanning behaviors, particularly the use of indoor tanning devices, are a likely contributing factor to skin cancer disparities. A past meta-analysis found that almost 90% of all melanomas, 85% of squamous cell carcinomas, and 82% of basal cell carcinomas were attributable to excess UV radiation exposure [6]. Indoor tanning devices expose users to high doses of radiation, making them dangerous enough that they are classified as a group I carcinogen [7]. Studies have shown that SGM males were 2.9–5.8 times more likely to have tanned indoors when compared to heterosexual males. Past studies found no difference in other UV-related risk factors between SGM samples and the general population, including outdoor sun exposure and infrequent sunscreen use [3]. Thus, it is likely that the use of indoor tanning devices contributes to the skin cancer disparity between these groups [4, 5].

Existing behavioral research focused on the use of indoor tanning devices in SGM populations is limited. In these studies, either gender was measured as a binary variable conflating sex at birth and gender identity or SGM subgroups were aggregated into one group, concealing variations across sexual and gender identities. SGM populations are heterogenous, and there are likely important psychosocial differences across SGM subgroups related to the use of indoor tanning devices. Appearance-based motivations have been found to be particularly important in cisgender gay and bisexual men, but this research was not diverse with regard to gender and sexual identities [8].

Problem behavior theory (PBT) posits that risk behaviors cluster because of latent personality characteristics that enable the behaviors, which are reinforced in the social environment [9]. Personality factors, such as sensation seeking and low inhibitory control, were associated with binge drinking and tobacco use in cisgender SGM men in previous research [10–12]. Based on PBT, we would expect that skin cancer risk behaviors, such as the use of indoor tanning devices, would correlate with other cancer risk behaviors found to be prevalent in SGM populations (e.g., tobacco and alcohol use); however, co-occurring cancer risk behaviors were not considered in previous studies on indoor tanning in SGM populations [5, 13, 14].

The Surgeon General's Call to Action to Prevent Skin Cancer was released in 2014, which outlined five strategic objectives for preventing skin cancer [15]. Among these, one was to equip individuals with the necessary knowledge to make informed and healthy decisions about their exposure to UV rays. A national study of the U.S. primary care providers found that over a quarter regularly counseled their patients on indoor tanning [16]. Given that SGM populations face barriers to accessing primary care [17], the role of primary care in ameliorating skin cancer disparities is significant.

The purpose of this study was to assess the use of indoor tanning devices among a heterogenous sample of SGM adolescents and adults. We sought to answer the following research questions: (RQ1) Which SGM subgroups were most likely to use indoor tanning devices? (RQ2) How was healthcare utilization associated with the use of indoor tanning devices? (RQ3) What was the association of cancer risk behaviors with the use of indoor tanning devices?

## 2. Methods

This was a secondary analysis of the 2020 LGBT Health Needs Assessment collected by the Pennsylvania Department of Health [18]. Respondents were SGM residents of Pennsylvania aged thirteen and older. They were recruited through a nonprobability community-based approach. Recruitment materials were distributed in English and Spanish by community organizations over a wide range of platforms, including e-mail, mailed postcards, websites, mobile phone applications, and social media. The 15-minute questionnaire was available in both Spanish and English. Inclusion criteria included responding yes to “Do you identify as LGBTQ?” and “Do you live in Pennsylvania?”

### 2.1. Measures

#### 2.1.1. Indoor Tanning and Skin Cancer History

Indoor tanning use was measured with the following item: “Not including spray-on tans, during the past 12 months, how many times have you used an indoor tanning device such as a sunlamp, tanning bed, or booth?” Responses were dichotomized to reflect any past 12-month indoor tanning. Participants were also asked the following question about their history of skin cancer: “At any time in your life, have you received a skin cancer diagnosis?” The response options were “Yes” or “No.”

#### 2.1.2. Sexual Orientation and Gender Identity (SOGI)

Current gender was determined using both sex at birth and current gender identity; for example, male sex at birth and current female was coded as transgender female and male sex at birth and current man was coded as cisgender male. The response options for sex at birth were “Male” and “Female,” and the response options for current gender identity included the following: “Man,” “Woman,” “Genderqueer,” “Nonbinary,” “Genderfluid,” and “Another gender.” Those who selected the last option were asked to fill in the blank to specify their gender identity.

Participants were asked, “Which of the following best describes your sexual orientation?” The response options were “Bisexual,” “Gay,” “Lesbian,” “Pansexual,” “Asexual,” “Demisexual,” “Queer,” or “Another sexual orientation.” Due to low cell sizes, “Asexual” and “Demisexual” were combined into the same category with “Another sexual orientation.”

#### 2.1.3. Cancer Risk Behaviors

Current smoking was assessed with a single item: “How often do you currently smoke cigarettes?” Responses were dichotomized as “not at all” compared to “some days” or “every day.” Binge drinking was assessed with a single item: “In the past 30 days, how often did you drink 5 or more alcoholic drinks in a day? (One drink is equivalent to a 12-ounce beer, a 5-ounce glass of wine, or a drink with one shot of liquor).” Responses were dichotomized as “never” compared to those reporting binge drinking at least once in the past 30 days.

#### 2.1.4. Sociodemographics

Demographic measures included current age in years, Hispanic or Latin ethnicity (no versus yes), race (“White,” “Black/African American,” “Asian,” “American Indian, Native American, or Alaskan native,” “Pacific Islander,” or “Another race”), and educational attainment. Participants also reported their county of residence, which was used to classify them as rural or urban based on the Center for Rural Pennsylvania's definition of population density.

### 2.2. Analyses

All analyses were conducted in SAS 9.4 (SAS Institute, Cary, NC). Bivariate differences were examined using chi-square test of independence. The following four hierarchical logistic regression models were estimated: unadjusted (model 1), demographics and SOGI (model 2), healthcare utilization (model 3), and the full model with the addition of cancer risk factors (model 4). Adjusted odds ratios with 95% confidence intervals were reported as the measure of association. List wise deletion was used for missing data on predictor variables (<2% for any given variable). Missing data resulted from participants exiting the survey before reaching the end and did not appear to be systematic for any one variable.

## 3. Results

Characteristics of the study sample are described in Table 1 (*N* = 5,192). Overall, 2.8% (*n* = 143) of participants had a previous skin cancer diagnosis. Past year indoor tanning was reported by 5.5% (*n* = 286) of participants with the usage rate by cisgender men being 9.5% (*n* = 4906). Bivariate correlates of indoor tanning were age, sexual identity, gender, binge drinking, and tobacco use (Table 1).

There were minimal differences in the unadjusted and adjusted models (Table 2). In the unadjusted models, all sexual identities were negatively associated with indoor tanning when compared to gay/lesbian; however, only queer identity (aOR = 0.39; 95% CI: 0.19–0.79) and being a transgender woman (aOR = 0.29; 95% CI: 0.11–0.82) remained significant in the fully adjusted model. Black race (aOR = 0.35; 95% CI: 0.14–0.88) and being <18 years old (aOR = 0.33; 95% CI: 0.13–0.85) were also negatively associated with indoor tanning in the adjusted models. After adjusting for all variables, the following were independently associated with indoor tanning: cisgender men (aOR = 1.79; 95% CI: 1.31–2.44), binge drinking (aOR = 1.94; 95% CI: 1.50–2.51), and tobacco use (aOR = 1.64; 95% CI: 1.21–2.21). Of note, the transgender male demographic was not associated with indoor tanning.

## 4. Discussion

Cisgender men showed the greatest prevalence of indoor tanning independent of sexual identity (RQ1), while indoor tanning was otherwise equivalent across sexual and gender identities with a few exceptions: queer-identified, nonbinary individuals, and transgender women were less likely to have used an indoor tanning device. Black respondents and those less than eighteen years of age were also less likely to have tanned indoors in the adjusted models. These findings add to existing research in a few important ways.

Participnts aged eighteen and younger had far lower rates of indoor tanning, suggesting that legal age restrictions may have their intended effects. In Pennsylvania, indoor tanning is prohibited for youth sixteen years and younger and parental consent is required for those seventeen years of age [19, 20]. Implementing this type of legislation in other states may help to reduce the use of indoor tanning devices among SGM youth more broadly.

The strong and independent association of cisgender male identity and indoor tanning device not only supports a targeted focus on this group for prevention [3] but also suggests that limiting inclusion to gay or bisexual identified men is not inclusive enough. A growing percentage of SGM youth is identified as pansexual (along with other identity labels) [21]. In this study, gender was predictive of indoor tanning regardless of sexual identity (e.g., gay, bisexual, pansexual, or other) with one exception (e.g., queer). Queer identified persons in this study were less likely to use indoor tanning devices. Future research should attempt to recruit cisgender males with diverse sexual identities to help elucidate these differences.

Recent binge drinking and tobacco use were also found to have a positive and independent association with indoor tanning. These findings support propositions from the Problem Behavior Theory [13, 14] and align with evidence from qualitative research that demonstrated how the social environment promotes tanning behaviors for many cisgender SGM men [8]. It also provides an opportunity for interventions. Addressing multiple risk factors simultaneously has long been shown to be more effective than addressing any one alone, since individuals participating in a given category of risky behaviors are likely to be participating in other risky behaviors as well [22]. Future research should investigate the specific intra- and interpersonal determinants of indoor tanning among cisgender SGM men across diverse sexual identities.

### 4.1. Implications

Overall, these findings suggest important implications for future intervention research. First, cisgender SGM males (inclusive of diverse sexual identities) should be targeted for intervention. Second, the null findings regarding healthcare utilization and prior skin cancer diagnosis suggest that patient-provider discussion regarding skin cancer prevention can be strengthened. Future research should determine if physicians are discussing the risks from indoor tanning devices with SGM patients and ways to improve SGM culturally responsive skin cancer risk communication. Lastly, given the association of indoor tanning with alcohol and tobacco use behaviors, physicians should consider screening for indoor tanning along with these other risk behaviors—particularly among SGM men. Focusing on co-occuring risk behaviors is especially important considering that alcohol and tobacco screenings are already standard in clinical practice and these behaviors are associated with indoor tanning in this study and in the general population [23].

### 4.2. Limitations

This study is limited in which the participants were recruited through nonprobability community-based sampling. Data were also self-reported, and a single measure of indoor tanning was used. As participants resided in Pennsylvania, results may not apply to the residents of other states. Also, measures of risk behaviors, personality, and social environment were limited given that this was a secondary analysis of existing data.

## 5. Conclusions

Cisgender SGM men are more likely to use indoor tanning devices compared to other SGM subpopulations. Because indoor tanning is associated with alcohol and tobacco use, screening for skin cancer risk behaviors should accompany standard tobacco and alcohol screenings in clinical practice.

## 6. Disclaimer

These data were supplied by Pennsylvania Department of Health, Bureau of Health Promotion and Risk Reduction, Division of Tobacco Prevention and Control, Harrisburg, Pennsylvania. The survey was conducted in collaboration with the Bradbury-Sullivan LGBT Community Center and the Research and Evaluation Group at Public Health Management Corporation. The Pennsylvania Department of Health specifically disclaims responsibility for any analyses, interpretations, or conclusions.

## Figures and Tables

**Table 1:** Sample characteristics stratified by sun protective behaviors (*N* = 5,192).

Variable	Total sample *N* (%)	Stratified by indoor tanning		Chi-square test of difference *p* value
		% Yes	% No	
Total		5.5	94.5	
Demographics				
Age				**<0.01**
13–18	451 (8.9)	2.0	98.0	
18–29	1778 (34.2)	4.6	95.4	
30–40	1238 (23.8)	7.2	92.8	
40+	1725 (33.2)	6.1	93.9	
County				0.50
Urban	4193 (81.8)	5.3	94.7	
Rural	936 (18.2)	5.9	94.1	
Race				0.08
White	4413 (85.4)	5.6	94.4	
Black/African American	210 (4.1)	2.4	97.6	
Asian	92 (1.8)	2.2	97.8	
Another race	455 (8.8)	6.5	9.5	
Hispanic				0.37
No	4841 (93.4)	5.4	94.6	
Yes	343 (6.6)	6.6	93.5	
Education level				0.25
Less than college	2389 (46.1)	5.1	94.9	
College or higher	2792 (53.9)	5.8	94.2	
SOGI				
Sex at birth				**<0.01**
Female	2831 (54.7)	3.4	96.6	
Male	2347 (45.3)	8.1	91.9	
Sexual orientation				**<0.01**
Gay/lesbian	2777 (53.6)	7.2	92.8	
Bisexual	1017 (19.6)	4.9	95.1	
Pansexual	468 (9.0)	3.9	96.1	
Queer	575 (11.1)	1.6	98.4	
Another sexual orientation	347 (6.7)	2.3	97.7	
Current gender				**<0.01**
Cisgender man	1874 (36.1)	9.5	90.5	
Cisgender woman	1802 (34.7)	4.2	95.8	
Transgender woman	293 (5.7)	2.1	97.9	
Transgender man	338 (6.5)	2.7	97.3	
Nonbinary	882 (17.0)	1.9	98.1	
Healthcare utilization				
Usual place of care				0.45
Yes	4219 (81.6)	5.4	94.6	
No	954 (18.4)	6.0	94.0	
Cancer risk				
Binge drinking (past 30 days)				**<0.01**
Yes	1789 (34.6)	8.9	91.1	
No	3387 (65.4)	3.7	96.3	
Tobacco cigarette user				**<0.01**
Yes	751 (14.5)	9.7	90.3	
No	4439 (85.5)	4.8	95.2	
Cancer history				
Previous skin cancer diagnosis				0.12
Yes	143 (2.8)	8.4	91.6	
No	5027 (97.2)	5.4	94.6	

*Note*. SOGI: sexual orientation and gender identity. There were minimal amounts of missing data so not all frequencies add up to 5,192. Bolded values are statistically significant, *p* < 0.05.

**Table 2:** Correlates of tanning bed use among sexual and gender minority adolescents and adults (*N* = 5,034).

Variable	Tanning bed use			
	Model 1 (unadjusted) OR (95% CI)	Model 2 (demographics + SOGI) aOR (95% CI)	Model 3 (demographics + SOGI + healthcare) aOR (95% CI)	Model 4 (demographics + SOGI + healthcare + cancer risk) aOR (95% CI)
Demographics				
Age				
13–18	**0.19 (0.08, 0.46)**	**0.28 (0.11, 0.71)**	**0.27 (0.11, 0.70)**	**0.33 (0.13, 0.85)**
18–29	0.77 (0.57, 1.03)	1.07 (0.78, 1.48)	1.04 (0.75, 1.44)	0.94 (0.67, 1.31)
30–40	1.18 (0.88, 1.59)	**1.46 (1.07, 1.98)**	**1.42 (1.04, 1.94)**	1.27 (0.92, 1.75)
40+	1.00	1.00	1.00	1.00
County				
Urban	0.89 (0.66, 1.20)	0.86 (0.63, 1.18)	0.86 (0.63, 1.18)	0.86 (0.62, 1.18)
Rural	1.00	1.00	1.00	1.00
Race				
White	1.00	1.00	1.00	1.00
Black/African American	0.44 (0.18, 1.07)	**0.38 (0.16, 0.95)**	**0.38 (0.15, 0.94)**	**0.35 (0.14, 0.88)**
Asian	0.40 (0.10–1.61)	0.40 (0.10, 1.66)	0.40 (0.10, 1.66)	0.46 (0.11, 1.90)
Another race	1.13 (0.75, 1.70)	1.19 (0.71, 2.01)	1.18 (0.70, 1.98)	1.15 (0.68, 1.94)
Hispanic				
No	1.00	1.00	1.00	1.00
Yes	1.31 (0.83, 2.05)	1.14 (0.65, 2.02)	1.14 (0.65, 2.00)	1.12 (0.64, 1.97)
Education level				
Less than college	1.00	1.00	1.00	1.00
College or higher	1.12 (0.88, 1.43)	0.91 (0.70, 1.18)	0.91 (0.70, 1.19)	0.97 (0.74, 1.26)
SOGI				
Sexual orientation				
Gay/lesbian	1.00	1.00	1.00	1.00
Bisexual	**0.68 (0.50, 0.94)**	1.07 (0.75, 1.52)	1.06 (0.74, 1.51)	1.08 (0.76, 1.55)
Pansexual	**0.44 (0.26, 0.75)**	0.88 (0.49, 1.59)	0.89 (0.50, 1.59)	0.88 (0.49, 1.56)
Queer	**0.21 (0.11, 0.41)**	**0.40 (0.20, 0.83)**	**0.40 (0.19, 0.82)**	**0.39 (0.19, 0.81)**
Other	**0.28 (0.13, 0.59)**	0.62 (0.27, 1.41)	0.62 (0.27, 1.41)	0.69 (0.30, 1.57)
Current gender				
Cisgender man	**2.31 (1.71, 3.05)**	**2.05 (1.51, 2.79)**	**2.05 (1.51, 2.79)**	**1.81 (1.32, 2.47)**
Cisgender woman	1.00	1.00	1.00	1.00
Transgender woman	**0.32 (0.12, 0.88)**	**0.30 (0.11, 0.83)**	**0.30 (0.11, 0.83)**	**0.29 (0.11, 0.82)**
Transgender man	0.61 (0.30, 1.23)	0.78 (0.38, 1.64)	0.80 (0.38, 1.66)	0.78 (0.37, 1.65)
Nonbinary	**0.40 (0.23, 0.70)**	**0.52 (0.29, 0.93)**	**0.51 (0.29, 0.93)**	**0.51 (0.28, 0.92)**
Healthcare utilization				
Usual place of care				
Yes	0.89 (0.66, 1.21)		0.85 (0.61, 1.17)	0.92 (0.67, 1.28)
No	1.00		1.00	1.00
Cancer risk				
Binge drinking (past 30 days)				
Yes	**2.44 (1.91, 3.12)**			**1.93 (1.49, 2.50)**
No	1.00			1.00
Tobacco cigarette user				
Yes	**2.11 (1.59, 2.81)**			**1.67 (1.23, 2.25)**
No	1.00			1.00
Cancer history				
Any previous cancer diagnosis				
Yes	1.42 (0.91, 2.21)			1.21 (0.63, 2.32)
No	1.00			1.00
Previous skin cancer diagnosis				
Yes	1.53 (0.81, 2.86)			1.09 (0.48, 2.49)
No	1.00			1.00

*Note*. OR: odds ratio; aOR: adjusted odds ratio; SOGI: sexual orientation and gender identity. Bolded values are statistically significant, *p* < 0.05.

## Data Availability

Data are publicly available at https://www.phmc.org/site/newsroom/press/108-press-releases/2021/1418-2020-pennsylvania-lgbtq-health-needs-assessment-findings-released.
